# Three-Dimensional Microstructural Properties of Nanofibrillated Cellulose Films

**DOI:** 10.3390/ijms15046423

**Published:** 2014-04-16

**Authors:** Arttu Miettinen, Gary Chinga-Carrasco, Markku Kataja

**Affiliations:** 1 Department of Physics, University of Jyväskylä, P.O. Box 35 (YFL) FI-40014 Jyväskylä, Finland; E-Mail: markku.t.kataja@jyu.fi; 2 Paper and Fibre Research Institute (PFI), Høgskoleringen 6 B, NO-7491 Trondheim, Norway; E-Mail: gary.chinga.carrasco@pfi.no

**Keywords:** nanofibrillated cellulose, NFC, oxygen transmission rate, OTR, tomography, humidity

## Abstract

Nanofibrillated cellulose (NFC) films have potential as oxygen barriers for, e.g., food packaging applications, but their use is limited by their hygroscopic characteristics. The three-dimensional microstructure of NFC films made of *Pinus radiata* (Radiata Pine) kraft pulp fibres has been assessed in this study, considering the structural development as a function of relative humidity (RH). The surface roughness, micro-porosity, thickness and their correlations were analyzed using X-ray microtomography (X–μCT) and computerized image analysis. The results are compared to those from scanning electron microscopy and laser profilometry. Based on a series of films having varying amounts of 2,2,6,6-tetramethylpiperidinyl-1-oxyl (TEMPO)-mediated oxidated nanofibrils, it was demonstrated that X–μCT is suitable for assessing the surface and bulk 3D microstructure of the cellulose films. Additionally, one of the series was assessed at varying humidity levels, using the non-destructive capabilities of X–μCT and a newly developed humidity chamber for in-situ characterization. The oxygen transmission rate (OTR) of the films (20 g/m^2^) was below 3.7mLm^−2^ day^−1^ at humidity levels below 60% RH. However, the OTR increased considerably to 12.4mLm^−2^ day^−1^ when the humidity level increased to 80% RH. The increase in OTR was attributed to a change of the film porosity, which was reflected as an increase in local thickness. Hence, the characterization techniques applied in this study shed more light on the structures of NFC films and how they are affected by varying humidity levels. It was demonstrated that in increasing relative humidity the films swelled and the oxygen barrier properties decreased.

## 1. Introduction

### 1.1. Nanofibrillated Cellulose

Vascular plant cell walls are composed of elementary fibrils which have been reported to have a width of 3.5nm [1,2]. The fibrils are composed of 36 *β*-1,4-glucan chains, which are synthesised by rosette complexes, *i.e.*, cellulose synthase proteins in the plasma membrane [3,4]. In wood, the fibrils are arranged differently in the different layers which compose the fibre wall, *i.e.*, the primary wall (P) and the secondary wall layers (S1, S2 and S3).

Nanofibrillated cellulose (NFC) refers to the material that is produced based on the disintegration of the fibre wall into a major fraction of individualized elementary fibrils and their aggregates. Chemical pretreatments such as TEMPO mediated oxidation yield homogenous fibrillated materials with typical fibril diameters less than 20nm (see also [5–7]). NFC can be produced from various green resources, such as wood, agricultural residues and annual crops [6–10], and is thus renewable and biodegradable. In addition, the material has several advantages from a mechanical, optical and structural point of view and has been proposed for several applications [6,11–16]. Films made of NFC have been reported to be strong (tensile strength > 200MPa, [17]) and with good oxygen barrier properties. For a good overview of oxygen permeability of NFC materials see [18], where values between 5 and 0.1mLμmm^−2^ day^−1^ kPa^−1^ have been reported (measured at 23 °C and 50% relative humidity), and also [13,19–22]. Additionally, NFC films have high translucency (>90% according to [19,21]), making them a suitable material for, e.g., food packaging applications. However, neat NFC films are hygroscopic, *i.e.*, the humidity reduces the oxygen barrier properties. An increase of the oxygen transmission rate (OTR) has been reported for NFC films when increasing the relative humidity (RH) (e.g., [21]). However, to our knowledge no direct assessment of the three-dimensional structural changes as a function of relative humidity has been reported.

### 1.2. Structural Quantification

It is important to perform an adequate quantification of the structure of NFC materials. From a structural point of view, thickness is an important property, which is utilized for estimating the tensile strength, density and oxygen permeability of NFC films. Additionally, multi-scale assessments of the surface roughness can yield detailed information of the surface structural properties of NFC films, which are affected by residual fibres and morphology of fibrils [23]. Structural characterisation tools such as scanning electron microscopy (SEM), laser profilometry (LP), atomic force microscopy (AFM) and transmission electron microscopy (TEM) have been successfully applied for quantification of NFC-based structures [7,8,19,23–29].

X-ray microtomography (X–μCT) is a non-destructive method to obtain the three-dimensional structure of a given sample [30]. Complemented by image analysis, it can be used to estimate, e.g., the thickness, roughness and micro-porosity of NFC films in a single measurement. Additionally, as the method is non-destructive, the same sample can be measured multiple times in varying environmental conditions. Thus it is relatively easy to obtain structural parameters as a function of, e.g., temperature or relative humidity.

It is the intention of this work to introduce the X–μCT characterization tool in combination with an in-situ equipment, which can be applied for assessing structural changes of hygroscopic materials such as NFC films. The suitability of these novel techniques is exemplified by performing a characterisation of the microstructure of NFC films, which have varying composition of TEMPO-mediated NFC as indicated in Table 1.

## 2. Results and Discussion

### 2.1. Structural Quantification

**Figure 1.**
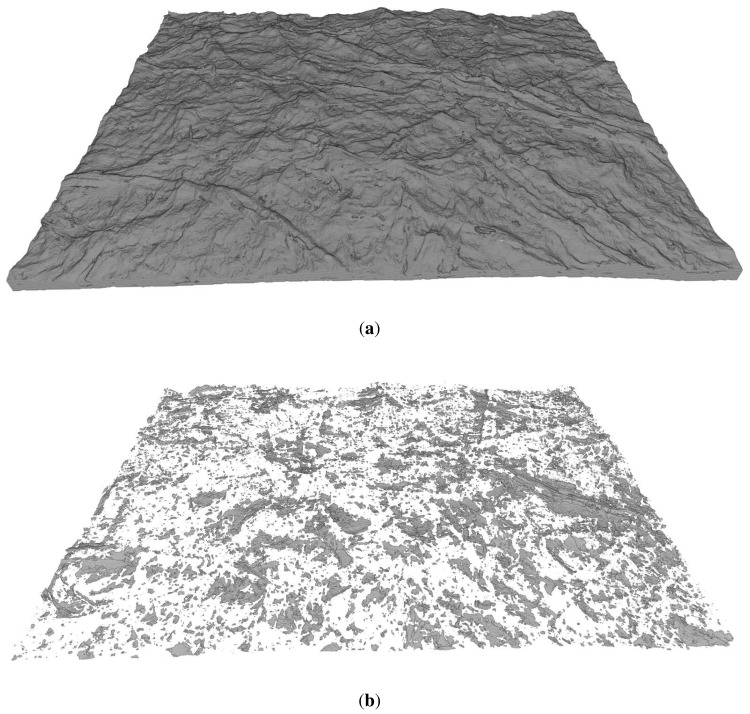
(a) Three-dimensional visualization of a tomographic image of film T0; (b) Similar visualization of micro-pores in the film T0. The dimensions of the film are approximately 1mm × 1mm × 15 μm. (30% RH, 23 °C).

**Figure 2.**
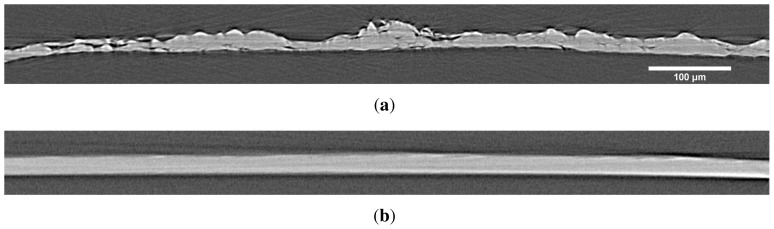
Single cross-sectional X–μCT slices of samples (a) T0 and (b) T100. Notice that the mean thickness seems to be approximately the same but surface roughness is different. There are also some pores visible in the T0. The thickness of the film in (b) is approximately 15 μm. (30% RH, 23 °C).

**Figure 3.**
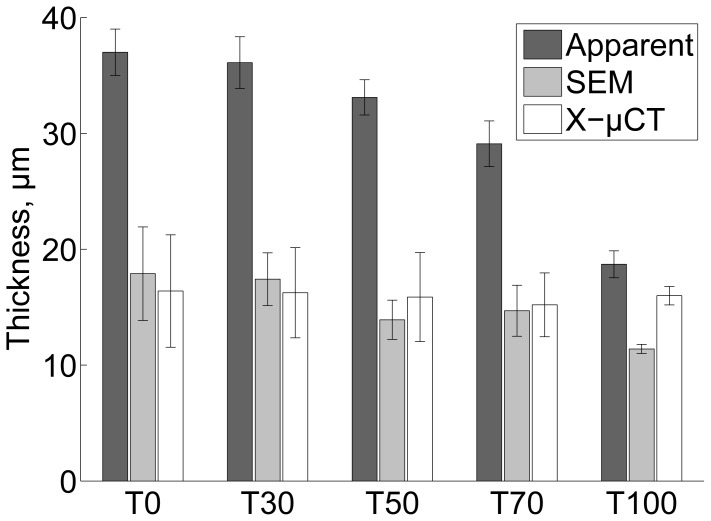
Comparison of film thickness determined using SEM and X–μCT (30% RH, 23 °C). The error bars are standard deviations over 10 replicates for apparent thickness and SEM thickness, 3 replicates for X–μCT measurements of T0 and T30 and 4 replicates for X–μCT measurements of T50, T70 and T100. The apparent thickness is determined using a micrometer, following ISO 534.

**Figure 4.**
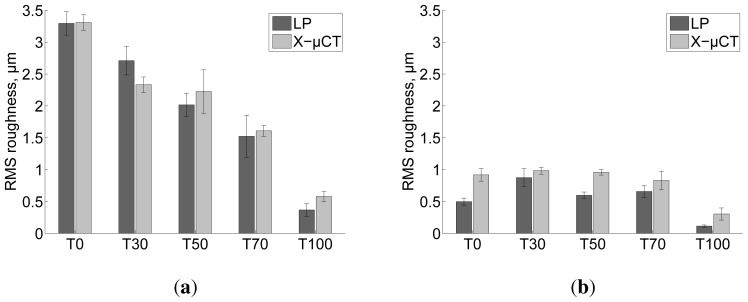
Comparison of (a) top surface and (b) bottom surface roughness determined using LP (50% RH, 23 °C) and X–μCT (30% RH, 23 °C). The error bars are standard deviations over 10, 3 and 4 replicates for LP measurements, X–μCT measurements of T0 and T30 and X–μCT measurements of T50, T70 and T100, respectively.

As an example of the X–μCT images, a three-dimensional visualization of film T0 is exemplified in Figure 1. Based on visual inspection, the film surface is very rough and the micro-porosity (pores of size scale ≳ 1 μm) contributes significantly to the total volume of the film. Figure 2 shows cross-sectional X–μCT slices of films T0 and T100. Based on the image, the mean thicknesses of the two films seem to be approximately equal, but the surface roughness of the T0 film is higher, especially in the top surface. In the T0 film there are also some pores that are not visible in the T100 sample. This observation is confirmed by the results shown in Figures 3 and 4, where the thickness and roughness of each individual film is presented. The thickness and roughness quantified by X–μCT are also compared with the corresponding results from SEM and LP respectively, which give similar estimates. It is worth to notice the large difference between the apparent thickness and the intrinsic thicknesses measured using SEM and X–μCT. As confirmed in this study, the thickness can be overestimated up to roughly 100%, depending on the NFC material and the device applied for thickness measurements (see also [29]).

**Figure 5.**
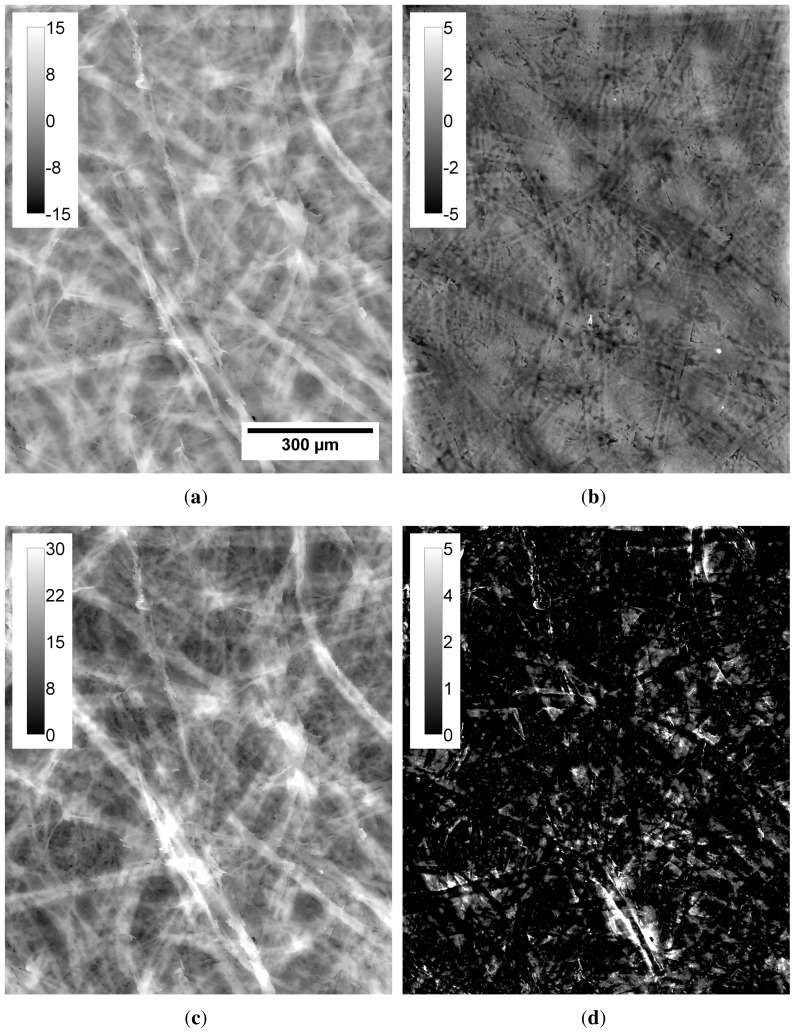
(a) Topography map of top surface; (b) topography map of bottom surface; (c) thickness map and (d) micro-porosity map of T0. Values in the calibration bars are given in micrometers. Width of each sub-image is 0.9mm. Notice high correlation between the topography of the top surface (a) and the thickness map (c). The micro-porosity in (d) seems to be located along the residual fibres. (30% RH, 23 °C).

**Figure 6.**
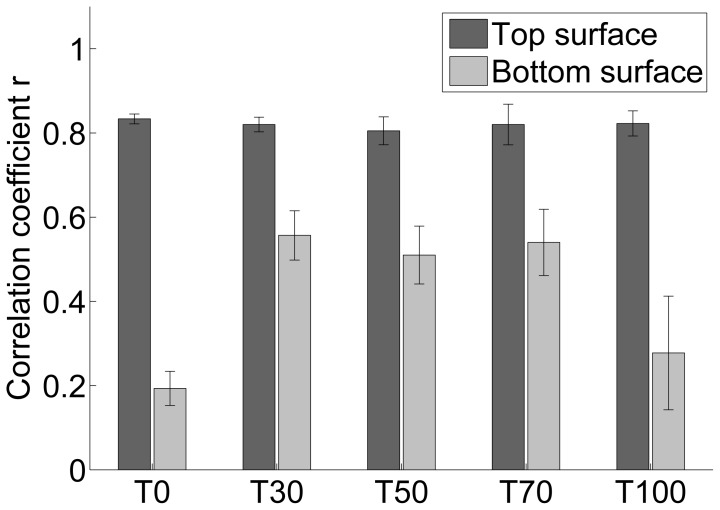
Correlation coefficient *r* between thickness and surface roughness. The error bars are standard deviations over 3 replicates for T0 and T30 and 4 replicates for T50, T70 and T100. (30% RH, 23 °C).

The surface topography, thickness and micro-porosity maps for film T0 are presented in Figure 5. Based on the maps, most of the thickness variations and roughness seems to be caused by residual fibres that form unevenness in the film surface. High correlation between top surface topography maps and thickness maps (Figure 6) shows that for all the films the top surface roughness is mostly related to variations in thickness. The roughness at the bottom surface, *i.e.*, the one that faced the petri dish surface, is low (Figure 4) and only weakly correlated with thickness (Figure 6). Recently it has been reported that the bottom surface of the films conforms to the surface of the petri dishes. The surface roughness of the petri dishes used also in this study has been quantified to be approximately 0.25 μm [23], corresponding approximately to the roughness of the bottom surface of sample T100.

**Figure 7.**
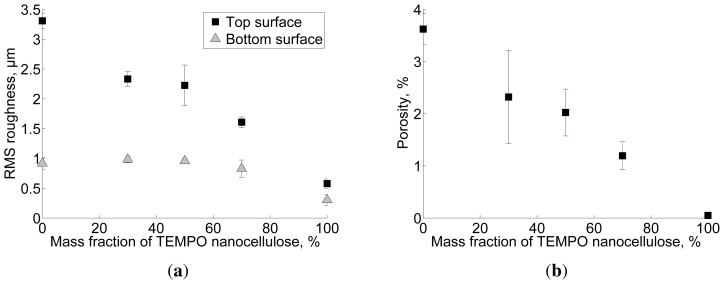
(a) Film roughness and (b) micro-porosity as a function of the mass fraction of TEMPO pretreated NFC. The error bars are standard deviations over 3 replicates for T0 and T30 and 4 replicates for T50, T70 and T100. (30% RH, 23 °C).

**Figure 8.**
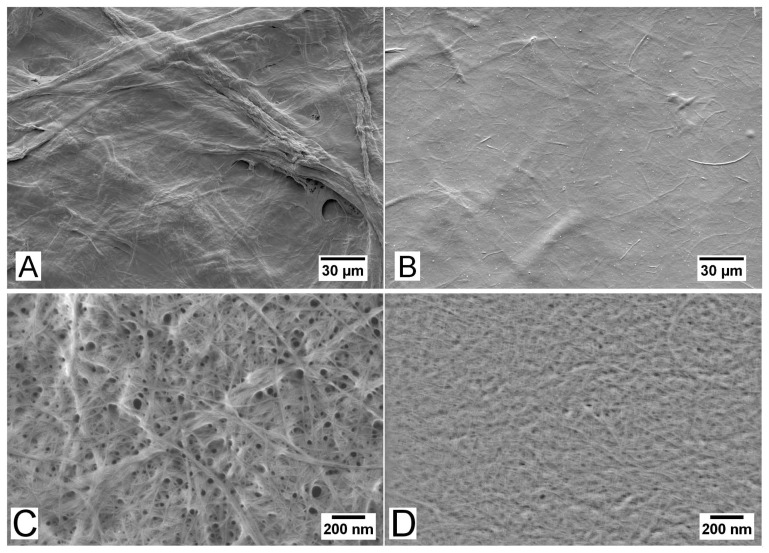
SEM images of the nanofibrillated cellulose samples (**A**,**C**) without and (**B**,**D**) with TEMPO mediated oxidation pre-treatment, corresponding to T0 and T100, respectively. The images in **C** and **D** exemplify the corresponding materials visualized at high magnification (50; 000×). For details on the image acquisition see [29].

In Figure 7a, where the surface roughnesses are plotted as a function of mass fraction of TEMPO pretreated NFC, the top and bottom surface roughnesses decrease when the mass fraction of TEMPO NFC increases. As described by [29] the NFC made of *P. radiata* pulp fibres and pretreated with TEMPO is highly fibrillated, containing mostly structures with diameter less than 20nm. The NFC manufactured without pre-treatment has a broader dimensional distribution, containing also fibres and fibre fragments (Figure 8; [5]). Hence, the characterization performed in this study confirms that the top surface roughness represents unevenness caused by residual fibres. Additionally, the effect of well-fibrillated TEMPO NFC on the micro-porosity of the corresponding films is demonstrated in Figure 7b. Higher fraction of TEMPO NFC leads to lower micro-porosity.

**Figure 9.**
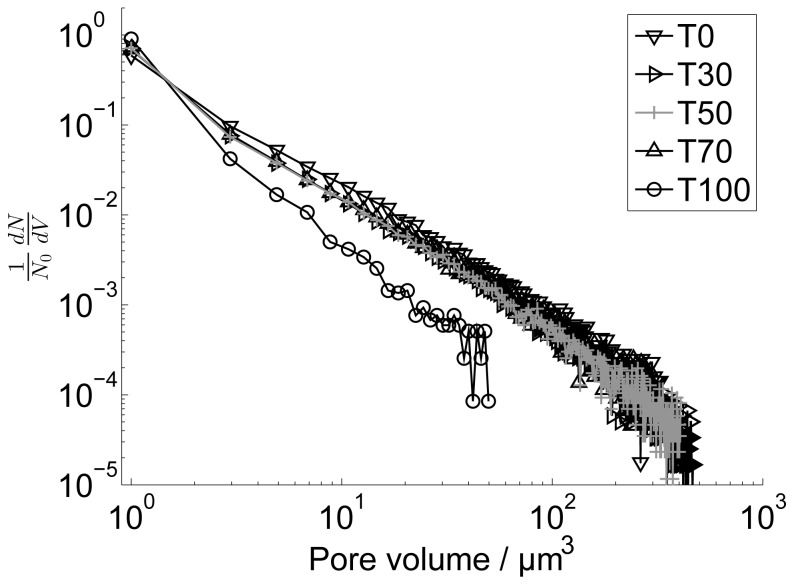
Pore volume distributions for the films as determined by X–μCT. (30% RH, 23 °C).

Although the quantified porosity seems to be rather low, it is important to note that the pores in the NFC films assessed in this study occur in a wide range of size scales, as indicated, e.g., by Figures 2 and 8. Figure 9 shows the part of the pore volume distribution within the micro-porosity range detectable using X–μCT. The increased scatter towards larger pore volume is related to the relatively low statistics and the selected bin size (≈ 2 μm^3^). On the other hand, the films contain also pores in smaller size scales, created by the random deposition of the nanofibrils (Figure 8). Considering the quantified intrinsic thickness (Figure 3), the density of the films is roughly between 1100 and 1400 kg/m^3^, which is in accordance with densities reported previously [22]. If it is assumed that the bulk density of NFC material is (1590±50) kg/m^3^ [35,36], a simple calculation shows that the total porosity is (21±4)% for all films in the present study. This suggests that only a small fraction of pores are in the micro-porosity range.

**Figure 10.**
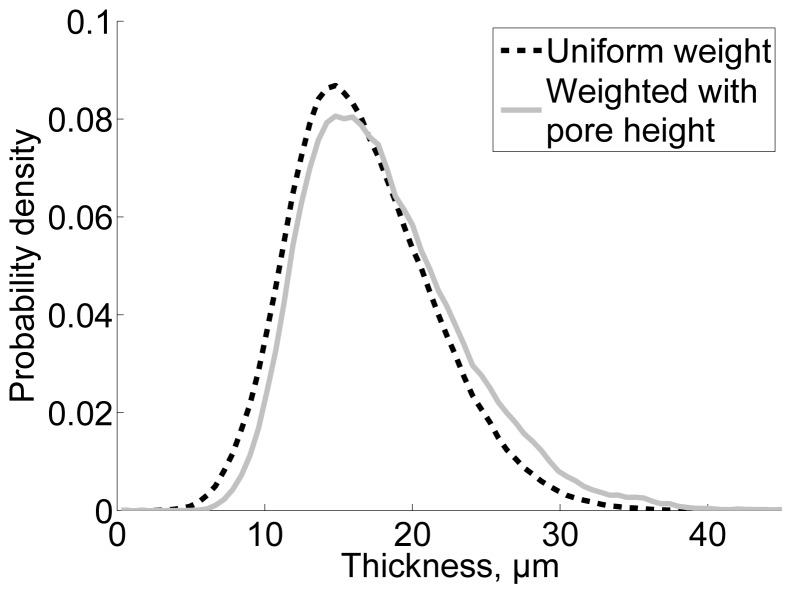
Thickness distribution of film area for T0. The black curve represents uniformly weighted distribution and the grey curve is a distribution weighted by local pore height. (30% RH, 23 °C).

Based on the micro-porosity map in Figure 5, most of the micro-pores seem to be found at locations along residual fibres. This observation is confirmed for film T0 (Figure 10). By comparing the uniformly weighted thickness distribution to the thickness distribution weighted by local pore height, it is demonstrated that more pores are located at the relatively thick parts of the film. Such thick areas have been previously attributed to locations containing residual fibres (Figure 5). Similar reasoning applies to the other samples assessed in this study. Similarly to the roughness, the micro-porosity decreases when the mass fraction of TEMPO pretreated NFC increases (Figure 7b). Based on the presented results, it can thus be concluded that also the micro-porosity, in addition to roughness, is mainly caused by residual fibres.

### 2.2. Oxygen Transmission Rate

**Figure 11.**
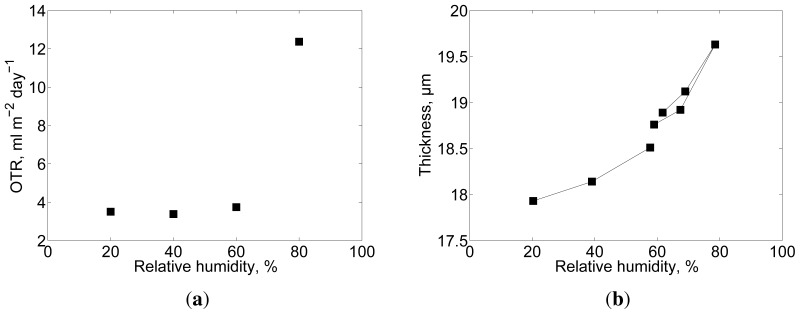
(a) Oxygen transmission rate and (b) thickness as a function of relative humidity for sample T30. The solid lines in (b) connect consecutive measurements, beginning from 20% RH and from 60% RH.

NFC films have been reported to be most adequate as barriers against oxygen [13,19,21,37], and thus the material has potential for food packaging applications. The OTR of sample T30 is approximately 3.7mLm^−2^ day^−1^ (measured at 60% RH, corresponding to oxygen permeability of 0.6mLμmm^−2^ day^−1^ kPa^−1^), which agrees with the OTR measured in [22] for 20 g/m^2^ films. There values of 4.12 and 3.68mLm^−2^ day^−1^ were determined for films similar to T0 and T100, respectively, measured at 50% RH. The relatively low OTR has been attributed to the high crystallinity of nanofibrils and the high fibrillation of the material, which facilitates the formation of dense film structures. However, cellulose is hygroscopic and the OTR of neat NFC films increases with increasing relative humidity [21,38,39]. This phenomenon is observed also in the films used in this work. At high relative humidity, water limits the hydrogen bonding between adjacent nanofibrils inducing an opening of the dense structures and thus reducing the packing of the fibrillated material [18]. Considering sample T30, this study exemplifies that there is a considerable increase in the OTR when the relative humidity increases to 80% (Figure 11a). It is important to emphasize that the OTR levels of the NFC films assessed in this study are comparable to ethylene vinyl alcohol (EVOH) (0.1–0.6mLμmm^−2^ day^−1^ kPa^−1^, 50% RH, 23 °C, [40]) and cellophane (3mLμmm^−2^ day^−1^ kPa^−1^, 50% RH, [38]). For a more detailed description of the barrier properties of various fibrillated materials see [18].

As shown in Figure 11b the film thickness increases significantly with increased humidity, confirming the swelling of the film. No hysteresis exceeding the scatter of the results near 60% RH is observable. Furthermore, no significant surface roughness or micro-porosity changes were quantified as a function of relative humidity.

### 2.3. Final Remarks

It is worth to emphasize that one of the advantages of using nanofibrillated cellulose is its renewability and biodegradability. Hence, NFC is a good alternative to, e.g., aluminium foil or petroleum based polymers, especially when it comes to barriers for food packaging. However, cellulose is hygroscopic and thus the films are affected by humidity. It is thus important to understand how the structure and barrier properties are affected by increased humidity, as we have demonstrated in this study. Moreover, due to the hygroscopic characteristics of NFC materials it is expected that the application of NFC in food packaging would require, e.g., addition of alternative components for reducing water intake and transmission. Some advances have been reported in this respect, considering also the grease resistance of the material (see [18] and references therein).

## 3. Experimental Section

### 3.1. Nanofibrillated Cellulose Films

Never dried *Pinus radiata* bleached market kraft pulp fibres were used in this study. The pulp fibres were homogenized with a Rannie 15 type 12.56X homogenizer, operated at 1000 bar pressure. The consistency of the pulp during homogenization was 0.5%. In addition, the bleached pulp fibres were pre-treated with TEMPO-mediated oxidation according to [9], and homogenized with the same conditions as above. The fibrillated materials were collected after 3 passes through the homogenizer. Five series of films of grammage 20 g/m^2^ were made by solution casting in petri dishes. The solid content of the NFC suspension was 0.2%. The series contained an increasing mass fraction of TEMPO pre-treated NFC, as described in Table 1.

A detailed quantification of the structural and chemical properties of the materials before and after homogenization has been done previously in [9] and [29], and is not repeated here. In the previous work it was noted that NFC manufactured without TEMPO pre-treatment is heterogeneous and contains a considerable amount of non-fibrillated fibres that are mostly absent in the TEMPO pre-treated NFC. The diameters of the fibrils in the TEMPO pre-treated material are thus mostly less than 20nm, whereas in the non-pre-treated case the diameter distribution is broader, extending to approximately 60nm (not counting the non-fibrillated fibres, [29]).

### 3.2. X-ray Microtomography

For X–μCT analysis, approximately 1mm × 4mm samples were cut and glued on top of a sample holder rod. X-ray microtomographic images of the samples were acquired using XRadia μCT-400 device (XRadia, Concord, CA, USA) with voxel size of 0.58 μm, resulting in imaged film volume of approximately 1mm^2^ × film thickness. The relative humidity and temperature during the X–μCT scans were approximately 30% and 23 °C, respectively. As the contrast-to-noise ratio between film and background was high (>10), the images were binarized using thresholding (see also Figure 2). The threshold value was decided using the Otsu method [31] and it was kept constant for all the images. After thresholding the bulk of the film was of value one whereas the background and the pores were zeroed. As voxels belonging to the film were mostly connected to each other, any artifacts were removed by zeroing all regions whose volume was less than 1000 voxels. Being located outside of the bulk of the film, the removal of the artifacts did not affect the detected porosity.

The surface topography maps were extracted for both surfaces, based on the borders between the binarized film and air. The acquired maps were processed with the ImageJ software using the SurfCharJ plugin [32]. The roughness described by the root-mean-square surface height *S_q_* was quantified at wavelengths less than 160 μm.

To facilitate the determination of pore size distribution, the binary image was inverted and voxels above the top surface and below the bottom surface were zeroed, leaving only the pores visible. The volume of each pore was calculated and the results were binned to yield the pore size distribution. A porosity map was also determined by considering the local height of pores corresponding to each pixel at the surface maps.

The thickness map of the film was determined as the local distance between the top and the bottom surfaces. The values of the thickness map were binned with uniform weight to yield the thickness distribution of film area. The binning was repeated using local pore height as weight to visualize the correlation between the local amount of pores and the thickness. Correlation coefficients between surface maps and thickness maps were determined using the CorrelationJ plugin [33]. From each material, at least 3 replicates were processed as indicated in Table 1.

**Figure 12.**
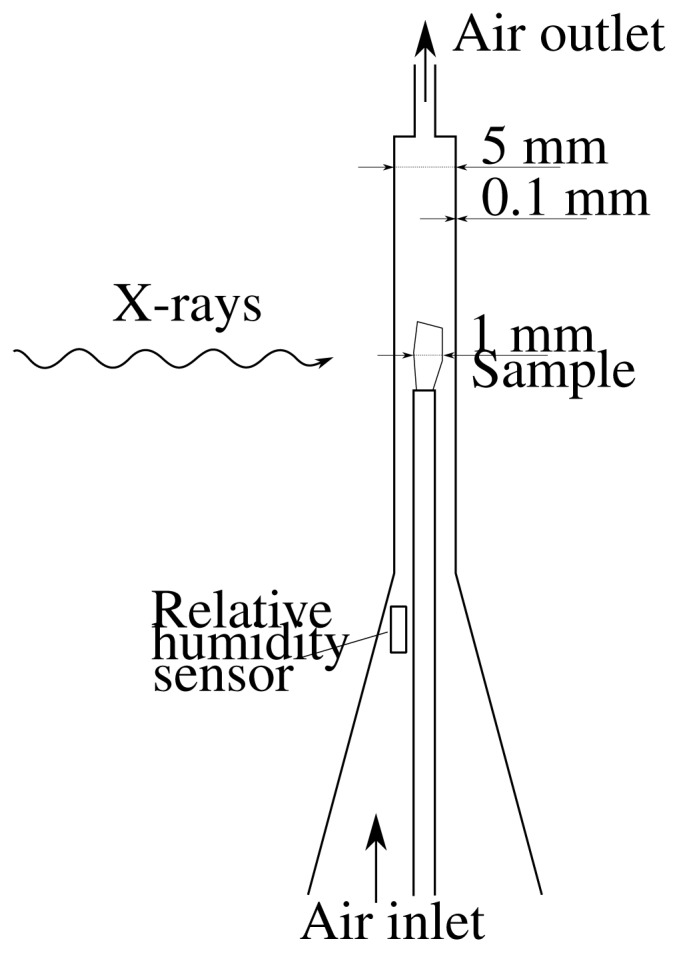
Schematic representation of the sample chamber used for humidity-controlled microtomographic scans.

To quantify the film structure in varying relative humidity, a special chamber was constructed, see Figure 12. The chamber was made of 0.1mm thick Polyether ether ketone (PEEK). PEEK was chosen as wall material as it is resistant to X-ray damage and has favourable machining properties. The chamber, containing the sample, was placed in the X–μCT device and air with predetermined relative humidity was constantly pumped through it using a HumiSys HF humidity generator (InstruQuest, Boca Raton, FL, USA). As the humidity generator and parts of required piping were located outside of a radiation protection cabinet, possibly in a different temperature from the sample, the relative humidity in the chamber was measured using a Sensirion SHT21 miniature sensor (Sensirion, Staefa, Switzerland). Before starting the X–μCT scan, the sample was let to stabilize for 4 hours. After the scan was finished, the relative humidity of the incoming air was adjusted to the desired level and the stabilization and the X–μCT scanning procedure was repeated. In the process the relative humidity was first cycled from 20% to 60%, where the scanning was paused for some days and the sample was returned to room humidity, approximately 30%. To reveal possible hysteresis in the structure and to quantify the precision of the measurement, the cycling was repeated from 60% RH to 80% RH and back to 60% RH.

X–μCT imaging process is very sensitive to instability and movement of the sample. As the X–μCT images didn’t contain any typical artifacts resulting from movement of the sample, we concluded that the four hour stabilization period was long enough. Being a time-consuming process, the sequence was performed for a single material only. Film T30 was chosen as it was expected to have high porosity but still being affected by the addition of TEMPO NFC. The resulting X–μCT images were processed similarly to those from non-humidity controlled scans.

### 3.3. Electron Microscopy

To visualize the surface of the films, pieces of samples T0 and T100 were assessed with scanning electron microscopy. Low magnification images (500×) were acquired with a Hitachi S-3000 variable pressure SEM (Hitachi High-Technologies Corporation, Minatoku, Tokyo, Japan), in secondary electron imaging mode. High-magnification images (50; 000×) were acquired from areas without gold coating, with a Zeiss Ultra field-emission SEM, using an inlens detector. The working distance and acceleration voltage were *<*1 mm and 0.5 kV, respectively. For details see [29].

For SEM cross-sectional analysis, pieces of 10mm × 20mm from each film were dried at 105 °C for 1 h and embedded in epoxy resin. The embedded samples were hand-held ground, and polished with polishing clothes of 9 and 1 μm. The prepared blocks were coated with a layer of carbon for SEM analysis. The microscope was a Hitachi S-3000 variable pressure SEM. Ten SEM cross-sectional images were acquired randomly from each film sample, applying the backscatter electron imaging (BEI) mode. The magnification was 500×, giving a resolution of 0.1 μm. The images were thresholded automatically according to Otsu [31]. The cross-sectional thickness was automatically quantified considering the upper and lower borders between the films and the epoxy resin.

### 3.4. Laser Profilometry

For laser profilometry (LP) analysis, samples of 10mm × 10mm were placed on an object glass with double-sided tape and coated with a layer of gold. Ten laser profilometry (Lehmann, Lehman Mess-Systeme AG, Baden-Dättwil, Germany) topography images were acquired from each sample. The size of the local areas was 1mm × 1mm, having a resolution of 1 μm/pixel. The LP image acquisition was performed at 50% RH and 23 °C. The acquired images were processed similarly to the surface topography maps extracted from the X–μCT images. The LP analysis was conducted on the top and on the bottom sides of the films.

### 3.5. Apparent Thickness

The apparent thickness of each film was measured using L & W Micrometer 51 from ten local areas, and the results were averaged to yield the apparent thickness for each film type. The thickness measurements were performed according to ISO 534 standard.

### 3.6. Oxygen Transmission Rate

The oxygen transmission rate (OTR) of sample T30 was measured with a Mocon OX-TRAN^®^ 1/50 at 20%, 40%, 60% and 80% relative humidity. The OTR of NFC films within the same series and measured at the same RH is relatively stable [22,34]. We have therefore undertaken only one OTR measurement for each humidity level.

## 4. Conclusions

X-ray computed tomography gives estimates comparable with LP and SEM for roughness and thickness of NFC films, respectively. An advantage of X-ray computed tomography is spatially isotropic resolution that allows analysis of micro-porosity and three-dimensional structural features. Based on a recently developed *in-situ* humidity chamber, the method allows also a robust control over environmental conditions during imaging, thereby enabling structural analyses of the same sample in different humidity conditions.

Additionally, the results of this study suggest that the fraction of residual fibres influences the micro-roughness and micro-porosity of NFC films. The large fraction of residual fibres encountered in sample T0 contributes to the formation of rough film structures, increasing the surface roughness and the micro-porosity. On the other hand, in sample T100 the residual fibres are absent, leading to smooth and dense structures.

## Figures and Tables

**Table 1.** Gravimetric composition of the NFC films.

Series	NFC (%)	TEMPO NFC (%)	Number of replicates in X–μCT
T0	100	0	3
T30	70	30	3
T50	50	50	4
T70	30	70	4
T100	0	100	4
